# A randomized, double-blind, placebo-controlled investigation of BCc1 nanomedicine effect on survival and quality of life in metastatic and non-metastatic gastric cancer patients

**DOI:** 10.1186/s12951-019-0484-0

**Published:** 2019-04-10

**Authors:** Maryam Hafizi, Somayeh Kalanaky, Hassan moaiery, Maryam Khayamzadeh, Sajad Noorian, Vahid Kaveh, Behrooz Gharib, Hossein Foudazi, Mohsen Razavi, Arash Jenabian, Saeid Salimi, Mohammad Mahdi Adib Sereshki, Hamid Reza Mirzaei, Afshin Zarghi, Saideh Fakharzadeh, Mohammad Hassan Nazaran, Mohammad Esmaeil Akbari

**Affiliations:** 1 Cancer Research Centre, Shahid Beheshti University of Medical Sciences, Tehran, Iran; 2 Department of Research and Development, Sodour Ahrar Shargh Company, Tehran, Iran; 3 Department of Statistics, Faculty of Sciences, University of Qom, Qom, Iran; 4 Firouzgar Hospital, Iran University of Medical Sciences, Tehran, Iran; 5 Naft Company Hospital, Tehran, Iran; 6 Shahid Fayaz-Bakhsh Hospital, Tehran, Iran; 7 Department of Medical Oncology and Hematology, Tehran Medical Sciences Branch, Islamic Azad University, Tehran, Iran; 8 Department of Medicinal and Pharmaceutical Chemistry, Shahid Beheshti University of Medical Sciences, Tehran, Iran

**Keywords:** BCc1 nanomedicine, Cancer, Gastric cancer, Clinical trial, Nanomedicine, Nanochelating technology

## Abstract

**Background:**

Currently, the main goal of cancer research is to increase longevity of patients suffering malignant cancers. The promising results of BCc1 in vitro and vivo experiments made us look into the effect of BCc1 nanomedicine on patients with cancer in a clinical trial.

**Methods:**

The present investigation was a randomized, double-blind, placebo-controlled, parallel, and multicenter study in which 123 patients (30-to-85-year-old men and women) with metastatic and non-metastatic gastric cancer, in two separate groups of BCc1 nanomedicine or placebo, were selected using a permuted block randomization method. For metastatic and non-metastatic patients, a daily dose of 3000 and 1500 mg was prescribed, respectively. Overall survival (OS) as the primary endpoint and quality of life (measured using QLQ-STO22) and adverse effects as the secondary endpoints were studied.

**Results:**

In metastatic patients, the median OS was significantly higher in BCc1 nanomedicine (174 days [95% confidence interval (CI) 82.37–265.62]) than in placebo (62 days [95% CI 0–153.42]); hazard ratio (HR): 0.5 [95% CI 0.25–0.98; p = 0.046]. In non-metastatic patients, the median OS was significantly higher in BCc1 nanomedicine (529 days [95% CI 393.245–664.75]) than in placebo (345 days [95% CI 134.85–555.14]); HR: 0.324 [95% CI 0.97–1.07; p = 0.066]. The QLQ-STO22 assessment showed a mean difference improvement of 3.25 and 2.29 (*p* value > 0.05) in BCc1 nanomedicine and a mean difference deterioration of − 4.42 and − 3 (p-value < 0.05) in placebo with metastatic and non-metastatic patients, respectively. No adverse effects were observed.

**Conclusion:**

The findings of this trial has provided evidence for the potential capacity of BCc1 nanomedicine for treatment of cancer.

*Trial registration* IRCTID, IRCT2017101935423N1. Registered on 19 October 2017, http://www.irct.ir/ IRCT2017101935423N1

**Electronic supplementary material:**

The online version of this article (10.1186/s12951-019-0484-0) contains supplementary material, which is available to authorized users.

## Background

Global statistics data in 2015 recorded 8.8 million deaths caused by cancer, which nearly equaled one-sixth of all deaths [1]. In 2018, 1,735,350 new cancer cases and 609,640 cancer deaths are calculated to happen in the US [2].

Among all cancers, gastric cancer has always been a major clinical challenge due to poor prognosis and inefficient treatments [3, 4]. Based on the National Cancer Database, in the United States, the overall 5-year (2008–2014) relative survival rate of patients having stomach cancer was about 31% [2], new stomach cancer cases were 7.2 per 100,000 people per year and the death toll was 3.2 per 100,000 people per year in 2011–2015 [5]. It is estimated that 26,240 people will be added to the previous gastric cancer patients in 2018, which will be 1.5% of all cancer patients [5].

Approximately half of the gastric cancer occurs in East Asian countries, showing a higher mortality rate than other countries [6]. Over half of gastric cancer deaths happen in the first year of diagnosis in Iran and another 30% during the second year of diagnosis. Like other developing countries, the results show lower survival rates in Iran [7].

Currently, radiotherapy, chemotherapy, surgery, or combinations of these are being used in cancer therapies, while every one of such treatments has both pros and cons [8]. Despite the preference for surgical resection treatment in patients with cancer, chemotherapy is considered the best available treatment for recurrent and advanced cancer patients who are not suitable for surgery [4].

Although chemotherapy is widely being used in clinics, it has also had dissatisfactory outcomes, which are mostly attributed to nonspecific drug distribution to healthy tissues, indiscriminate destruction of normal cells, toxicity of conventional chemotherapeutic drugs and the capability of cancerous cells to pump out the drug using multidrug resistance mechanisms along with heterogeneity of cancer [9, 10].

Nanotechnology has recently revolutionized chemotherapy and cancer treatments [11]. Nanomedicine can be more effective in treatment of cancer by targeting cellular characteristics of solid tumors. Using nanomedicine in treatments can reduce the resistant clonal population of cancerous cells [12, 13], so in recent years, more nanomedicine drugs targeting cancer cells are being nominated to be approved by Food and Drug Administration (FDA) [10]. Having said that, nanomedicine formulations need to be revised in terms of efficacy [14].

Nanochelating is a mother technology [15] providing a high-yield technique with a wide range of scientific and technological applications [16, 17] by synthesizing nanostructures via self-assembly [18] and bottom-up method. Such nanostructures are smart molecules showing favorable reactions according to the physicochemical conditions of the environment, which will subsequently modulate the balance of contained elements in cellular microenvironments based on their designed nanostructure [19].

In our previous study, we used this technology to synthesize BCc1 [8], having chelating properties with dominant affinity for iron element. Also, we evaluated the anticancer effects of BCc1 in vivo and vitro studies.

We observed apoptosis inducing effects on cancer cells, while BCc1 had no negative effects on normal cells at the same concentration and also showed protecting effects against oxidative stress.

After considering intraperitoneal (IP) lethal dose (LD50) of BCc1, it was revealed that BCc1 increased survival in mice bearing cancer [8].

In continuation of our preclinical studies, due to short longevity of patients with gastric cancer, we tested this nanostructure on patients suffering from gastric cancer [4, 20]. This investigation was conducted in the form of a randomized, double-blind, placebo-controlled study in two metastatic and non-metastatic gastric cancer groups to assess the overall survival (OS) and quality of life (QOL) in each. Along the study, any probable side effect pertained to the prescribed drug was also taken into account.

## Methods

### Trial design

The purpose of this study was comparing BCc1 nanomedicine and placebo groups.

The study was designed based on two main groups (metastatic and non-metastatic gastric cancer) that were separately divided into two sub-groups (drug and placebo).

A randomized, double-blind, placebo-controlled, and parallel trial was conducted at Cancer Research Center of Shahid Beheshti University of Medical Sciences.

Patients were collected from the oncology center of Shohadaye Tajrish Hospital, Bu Ali, Naft Company, Imam Reza, Firouzgar, and Shahid Fayaz Bakhsh hospitals in Tehran, Iran.

All groups received BCc1 nanomedicine or placebo capsule in addition to the base treatment, so it didn’t interfere in physician’s protocol. The analysis report was conducted after an 18-month follow-up.

### Participants

#### Recruitment, randomization, and allocation

Patients with adenocarcinoma gastric cancer were first invited to participate in the present study with a prior notice and then their eligibility criteria were evaluated by the researcher in charge.

Patients, the clinician, and the researcher in charge were blinded to treatment allocation. The patients were assigned to BCc1 nanomedicine and placebo groups based on the blocked randomization form [21].

#### Sample collection

Detailed trial information was all explained to patients by the researcher in charge and written informed consent was taken from all patients before enrollment. After that, all patients, already registered at Cancer Research Center of Shahid Beheshti University of Medical Sciences, were visited by the clinician, and then following the confirmation of their metastatic or non-metastatic gastric cancer using their medical records including endoscopic ultrasound (EUS), computed tomography scan (CT scan), and positron emission tomography scan (PET scan), they were sent into one of the main groups.

Finally, grouping classifications for clinical (c-stage) and post-neoadjuvant treatment (ypStage) stages for gastric adenocarcinoma were proposed for the eighth edition of the American Joint Committee on Cancer (AJCC) staging system guideline [22].

During the study, the patients who underwent a surgery and were unable to swallow did not take any drugs or placebo for 3 weeks.

### Inclusion criteria

- Men and women aged 25–85.
- Patients who were able to swallow.
- Patients confirmed with adenocarcinoma gastric cancer including gastroesophageal junction.
- Patients suffering metastatic and non-metastatic gastric cancer signing the consent form.

### Exclusion criteria

- Patients who declined to continue taking medicine.
- Observing adverse symptoms such as unacceptable toxicity in the patients after taking the medicine.

### Withdrawal criteria

The patients were free to withdraw from the study at any time without any need to provide the reason, but they allowed the continuation of data collection.

### Primary outcome measurement

- The primary main goal of the present study was OS. This period was measured from randomization time to death, irrespective of the cause.

### Secondary outcome measurement

- *Quality of life* The secondary main goal included QOL according to the European Organization for Research and Treatment of Cancer (QLQ-ST22) questionnaire [23] once before the treatment and once after 1.5 months for patients with metastatic gastric cancer and 4 months for patients with non-metastatic gastric cancer.
- *Adverse effect* Adverse effects including vomiting, nausea, neutropenia, anorexia, vital signs and hematological and biochemical tests in blood were assessed and recorded in all patients.

### Sample size

63 metastatic gastric cancer patients, out of whom 33 received BCc1 nanomedicine and 30 received placebo, were recruited randomly for the present study. In addition, 60 non-metastatic gastric cancer patients, out of whom 30 received BCc1 nanomedicine and 30 received placebo, were recruited randomly.

### Follow-up visits

All patients were followed up on phone calls on a weekly basis and were also visited by the clinician repeatedly. In addition, patients were free to consult with the researcher in charge for any reason at any time, including the occurrence of an adverse event.

### Data collection

The researcher in charge collected the information and checked for the missing values and consistency in patients during the study. Full details of data management procedures are available at any time.

### Intervention

Both BCc1 and placebo capsules were exactly identical in terms of shape and size. The treatment doses were specified according to “Guidance for Industry Estimating the Maximum Safe Starting Dose in Initial Clinical Trials for Therapeutics in Adult Healthy Volunteers” guideline [24].

In the previous animal study, we obtained LD50 for BCc1 nanomedicne (Additional file 1) by which we acquired No Observed Adverse Effect Level (NOAEL) for clinical study. The most famous and applicable method to adjust effective animal dose to human dose is calculating the effective animal dose for body surface area based on mg/m^2^. Therefore, we considered LD50 the reference to normalize the effective dose and then calculated it based on mg/m^2^, we chose the lowest dose at the safe range as first-in-human (FIH) for non-metastatic patients and highest dose at the safe range as FIH for metastatic patients [24].

A daily dose of 3000 mg was used for metastatic patients and 1500 mg for non-metastatic patients at three servings. The patients were monthly provided with medicine free of charge, while the researcher in charge became sure the patients took the medicine in the previous months.

Active pharmaceutical ingredient (API) synthesis of BCc1 nanomedicine was done in Sodour Ahrar Shargh Company’s laboratory using nanochelating technology [15] and then it was capsulized in Tehran Darou Pharmaceutical Co. in Tehran, Iran. Yet, both synthesizing and capsulizing placebo API were done in Tehran Darou Pharmaceutical Co. in Tehran, Iran.

### Statistical methods

The Kaplan–Meier [25] and Lifetime Table were used for construction of survival curves, and comparisons were performed using the log-rank test [26]. Hazard ratios (HRs) and 95% confidence interval (CI) were derived from cox proportional hazards models stratified by the two randomization stratification factors [27].

For QOL, first, the mean scores for all patients were calculated and then compared between the two groups (BCc1 nanomedicine and placebo) with Paired Samples T-Test (parametric statistic) and Wilcoxon Signed Ranks Test (non-parametric statistic) used for statistical comparison. A p-value of ≤ 0.05 was considered significant for covariate selection. All analyses were conducted using SPSS software (version 25; SPSS Inc., Chicago, IL, USA) [28].

## Results

### Patients’ disposition and characteristics

The patients were recruited between October 26, 2016 and May 19, 2018. The average ages for metastatic BCc1 nanomedicine and placebo groups at stage IV were 59.8 ± 13 and 61.2 ± 12.93, respectively, and in non-metastatic BCc1 and placebo groups at stages I, II, and III the average ages were 65 ± 10.5 and 61 ± 11.4, respectively.

**Fig. 1:**
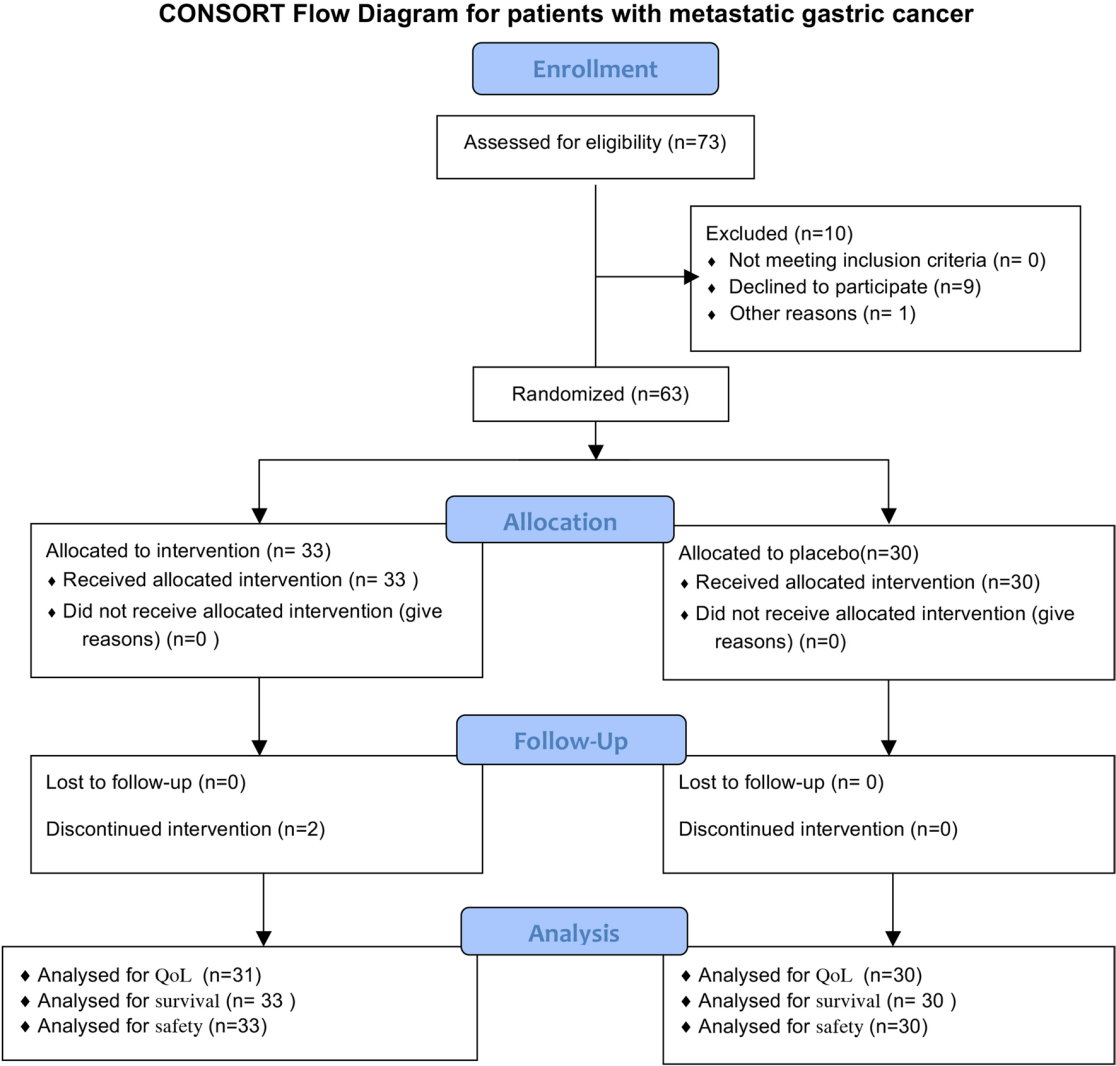
Consort flow diagram for patients with metastatic gastric cancer

**Fig. 2:**
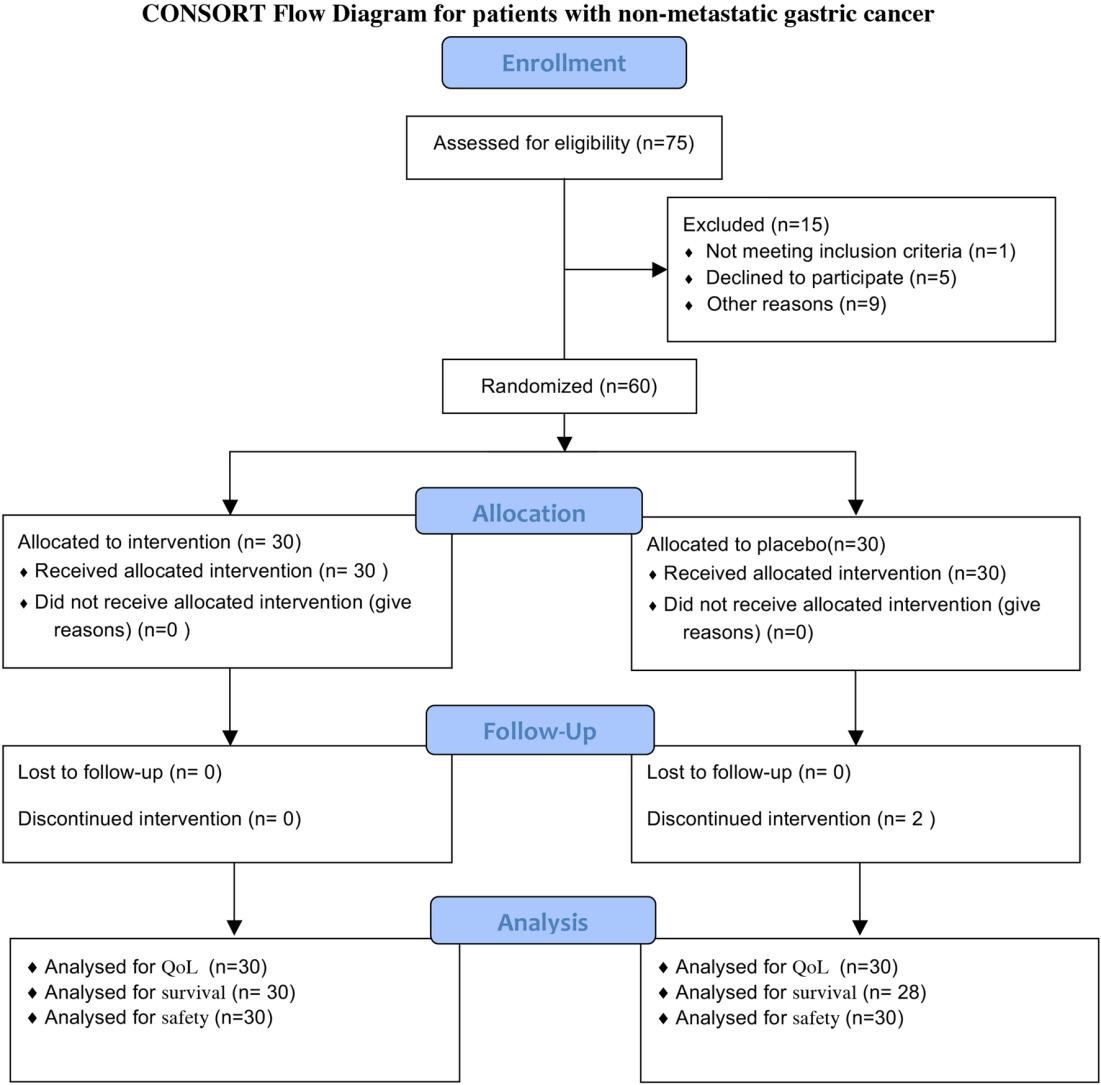
Consort flow diagram for patients with non-metastatic gastric cancer

As shown in Figs. 1 and 2, the number of all patients who were initially recruited on a random basis was 148, out of whom 25 were excluded from the study because of ineligibility, incomplete histological confirmation, among other reasons (These tables are prepared according to Consolidated Standards of Reporting Trials Form) [29]. The metastatic and non-metastatic patients’ characteristics are shown in Tables 1 and 2.

**Table 1:** Patients’ characteristics with metastatic gastric cancer

	BCc1 nanomedicine (n = 33)	Placebo (n = 30)
Age, years		
Mean (SD)	59.8 ± 13	61.2 ± 12.93
Weight		
Mean (SD)	61.68 ± 12.24	55.62 ± 12.82
Gender (n)		
Male–female	23–10	20–10
Metastatic site (n)		
Liver	17	11
Multiple site	9	10
Lung	2	3
Peritoneal carcinomatosis	5	4
Stage 4 (n)	33	30
Site of involvement (n)		
Cardia	7	10
Antrum	6	5
Fundus	3	3
Greater curvature	3	4
Lesser curvature	4	3
Diffuse	4	5
Intervention + chemotherapy (n)	14	19
Intervention + chemotherapy + radiotherapy (n)	0	1

**Table 2:** Patients’ characteristics with non-metastatic gastric cancer

	BCc1 nanomedicine (n = 30)	Placebo (n = 30)
Age, years		
Mean (SD)	65 ± 10.5	61 ± 11.4
Weight		
Mean (SD)	62 ± 14	59 ± 11
Gender (n)		
Male–female	20–10	14–19
Stage (n)		
I	3	1
II	3	8
III	19	13
Site of involvement (n)		
Cardia	10	9
Antrum	9	6
Fundus	0	3
Greater curvature	3	1
Lesser curvature	7	5
Diffuse	2	2
Intervention + chemotherapy (n)	12	20
Intervention chemotherapy + radiotherapy (n)	1	2

### Overall survival

**Fig. 3:**
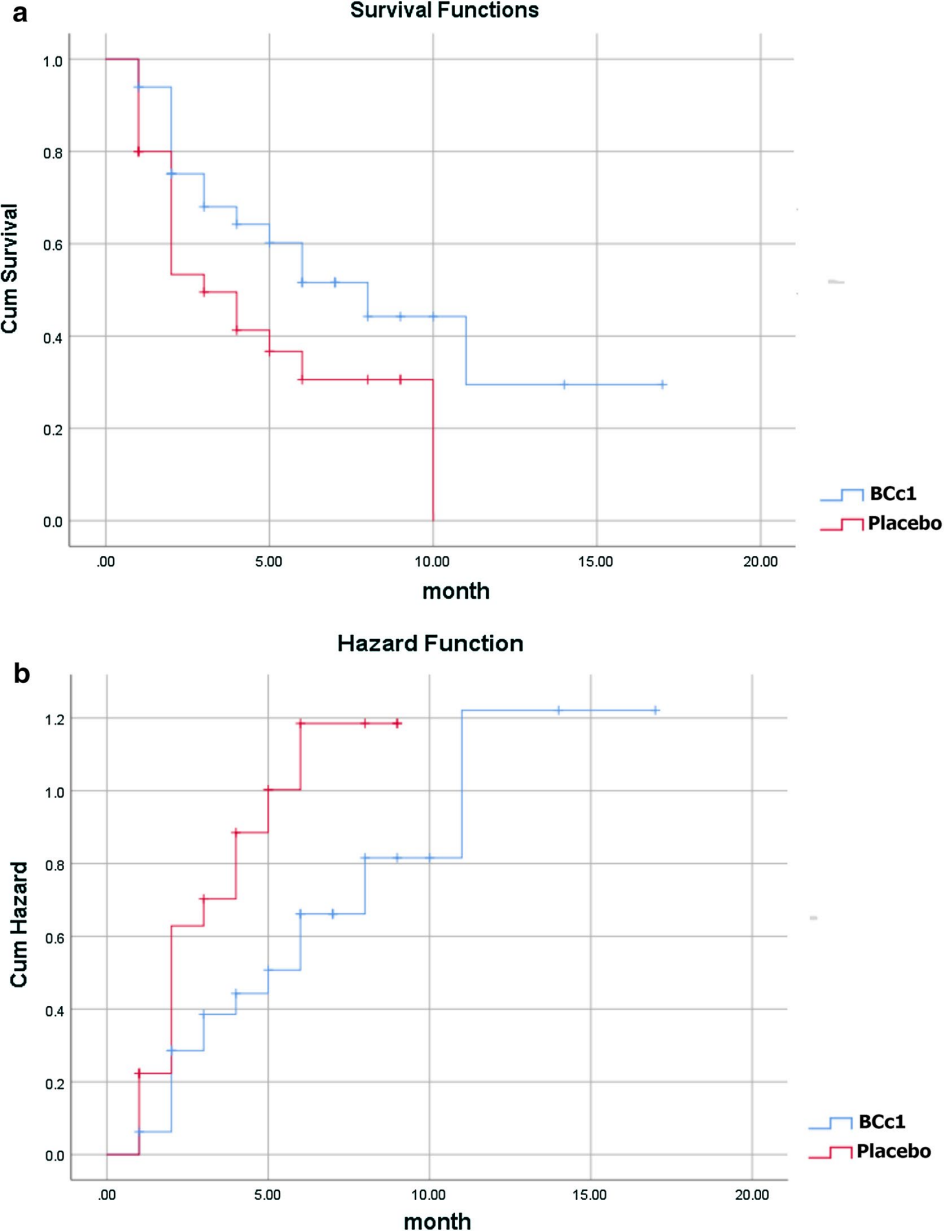
**a** Kaplan–Meier diagram of survival. **b** Hazard function diagram in metastatic patients (Lifetime tables of hazard and survival for patients who received BCc1 nanomedicine show a higher survival and lower hazard than patients who received placebo)

As shown in Table 3A, in metastatic patients, the median OS in BCc1 nanomedicine and placebo patients was 174 [95% CI 82.37–265] and 62 days [95% CI 0–153.42], respectively, thus the results showed that in metastatic patients who took BCc1 capsules, the median OS was 112 days significantly higher than that of patients who took placebo capsules; HR: 0.5 [95% CI 0.25–0.98; p = 0.046]. Kaplan–Meier diagram of survival and hazard function diagram of metastatic patients are shown in Fig. 3a, b.

**Table 3:** (A) Median of overall survival analysis in all metastatic and non-metastatic gastric cancer patients in the whole study. (B) Death percentage analysis in all metastatic and non-metastatic gastric cancer patients in the whole study

Group	Metastatic		Non-metastatic	
Total number	BCc1 (n = 33)	Placebo (n = 30)	BCc1 (n = 30)	Placebo (n = 30)
*(A) Median of overall survival analysis*				
Median (days)	174	62	529	345
Std. error	46.748	46.643	69.263	107.218
Median 95% CI (lower)	82.374	0.000	393.245	134.854
Median 95% CI (upper)	265.626	153.421	664.755	555.146
P-value for test of equality of survival distributions	0.04		0.05	
HR (BCc1/Placebo)	0.5		0.324	
*(B) Death percentage analysis*				
Death percentage	48.5%	63.3%	12.9%	29.6%

Test of equality of survival distributions (for Bcc1 and placebo groups) assay with Log Rank (Mantel-Cox)

**Fig. 4:**
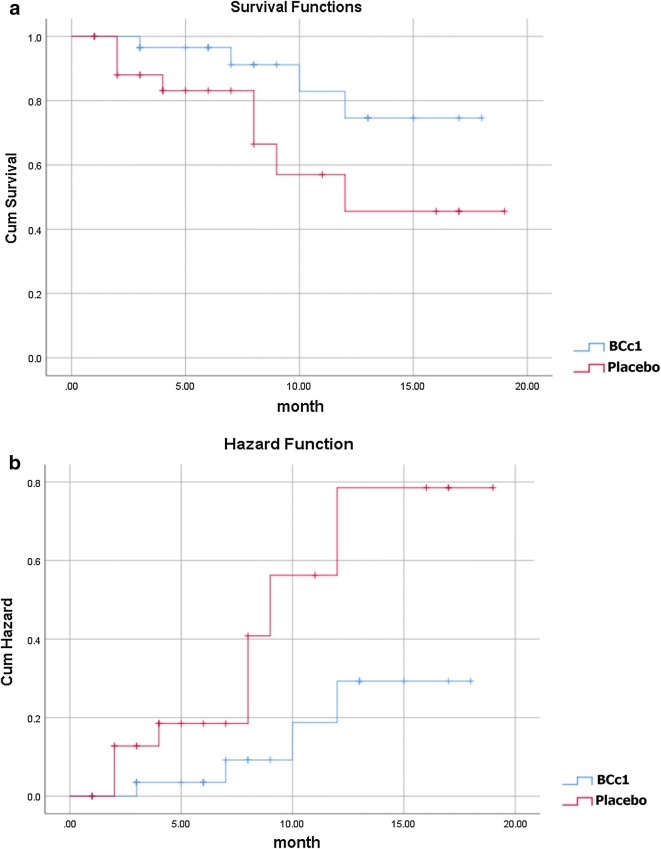
**a** Kaplan–Meier diagram of survival. **b** Hazard function diagram in non-metastatic patients (Lifetime tables of hazard and survival for patients who received BCc1 nanomedicine show a higher survival and lower hazard than patients who received placebo)

Likewise, as shown in Table 3A, in non-metastatic patients the median OS in BCc1 and placebo patients was 529 [95% CI 393–664] and 345 days [95% CI 134.85–555.147], respectively, thus the results showed that in non-metastatic patients who took BCc1 capsules, the median OS was 184 days significantly higher than that of patients who took placebo capsules; HR: 0.324 [95% CI 0.97–1.07; p = 0.066]. Kaplan–Meier diagram of survival and hazard function diagram of non-metastatic patients are shown in Fig. 4a, b.

As shown in Table 3B, in metastatic patients, 48.5% of patients in BCc1 nanomedicine group and 63.3% in placebo group, and in non-metastatic patients, 12.9% in BCc1 nanomedicine group and 29.6% in placebo group died during the study.

In the same study, we did an analysis to investigate patients’ OS who received BCc1 nanomedicine or placebo and chemotherapy (three types of regimens FOLFOX, DCF, and FLOT) simultaneously.

**Fig. 5:**
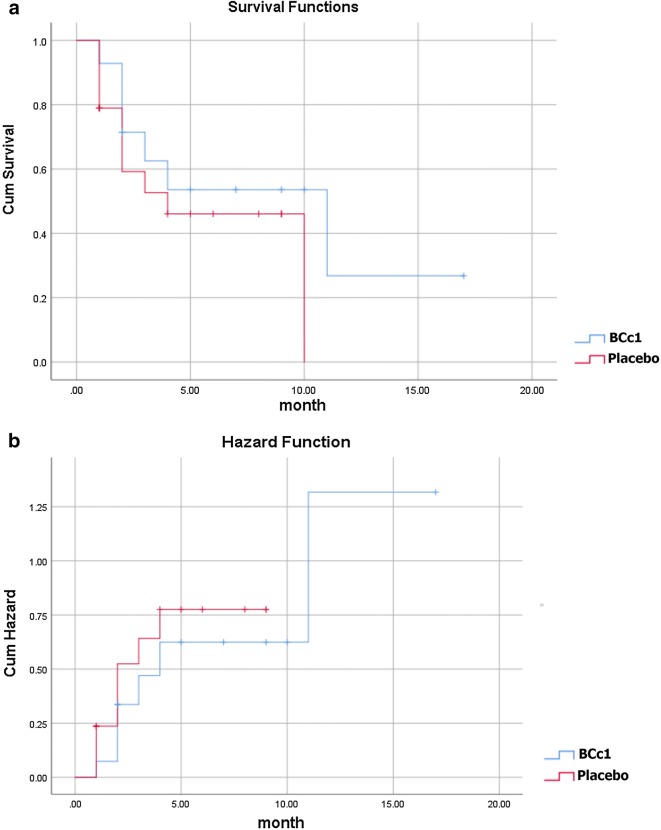
**a** Kaplan–Meier diagram of survival. **b** Hazard function diagram in metastatic patients who received intervention (BCc1nanomedicine or placebo capsule) and chemotherapy simultaneously

As shown in Table 4A, in metastatic patients, the median OS in BCc1 and placebo patients was 302 [95% CI 40.87–563.13] and 107 days [95% CI 0–214.98], respectively, while they underwent chemotherapy at the same time. The results revealed that in metastatic patients who took BCc1 capsules and simultaneously underwent chemotherapy, the median OS was 195 days longer, although not significantly, than the median OS of those who received placebo capsules and chemotherapy at the same time; HR: 0.589 [95% CI 0.212–1.64; p = 0.311]. Kaplan–Meier diagram of survival and hazard function diagram of metastatic patients who received BCc1 nanomedicine or placebo and underwent chemotherapy at the same time are shown in Fig. 5a, b.

**Table 4:** (A) Analysis of median of overall survival received interventions (BCc1nanomedicine or placebo) and chemotherapy simultaneously. (B) Death rate analysis received interventions (BCc1nanomedicine or placebo) and chemotherapy simultaneously

Group	Metastatic		Non- metastatic	
Total number	BCc1(n = 14)	Placebo (n = 19)	BCc1(n = 12)	Placebo (n = 20)
*(A) Median of overall survival analysis*				
Median (days)	302	107	482	265
Std. error	133.23	55.09	–^a^	27.769
Median 95% CI (lower)	40.87	0	–^a^	210.573
Median 95% CI (upper)	563.13	214.98	–^a^	319.427
P-value for test of equality of survival distributions	0.306		0.019	
HR^b^ (BCc1/Placebo)	0.589		0.019	
Stage (n)				
I	0	0	0	0
II	0	0	0	3
III	0	0	11	10
IIII	14	19	0	0
*(B) Death rate analysis*				
Death rate (%)	50	53	0	35

Test of equality of survival distributions (for Bcc1 and placebo groups) assay with Log Rank (Mantel-Cox)

^a^No statistics are computed because all cases are censored

^b^Univariate cox regression model

**Fig. 6:**
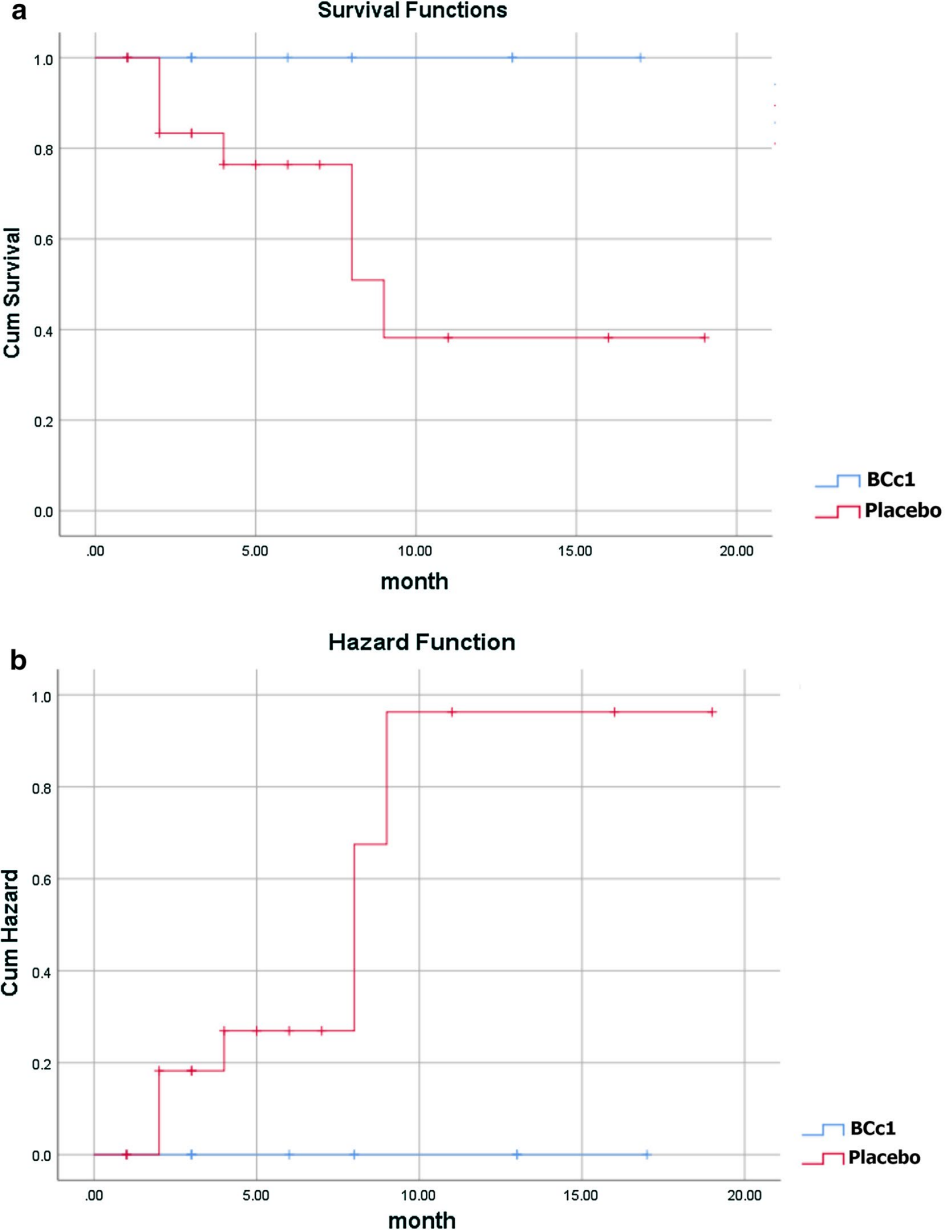
**a** Kaplan–Meier diagram of survival. **b** Hazard function diagram in non-metastatic patients who received intervention (BCc1nanomedicine or placebo capsule) and chemotherapy simultaneously

However, as shown in Table 4A, in non-metastatic patients, the median OS in BCc1 and placebo patients was 482 and 265 days [95% CI 210.57–319.42], respectively, thus in non-metastatic patients who took BCc1 capsules and simultaneously underwent chemotherapy, the median OS was 217 days significantly longer than the median OS of those who received placebo and chemotherapy at the same time; HR: 0.019 [95% CI 0–0.065; p = 0.207]. Kaplan–Meier diagram of survival and hazard function diagram of non-metastatic patients who received the interventions (BCc1 nanomedicine or placebo) and underwent chemotherapy at the same time are shown in Fig. 6a, b.

As shown in Table 4B, in metastatic patients, 50% of patients in BCc1 nanomedicine and 53% in placebo, and in non-metastatic patients, no patients in BCc1 nanomedicine and 35% in placebo died during the study, while patients of these groups received the interventions (BCc1 nanomedicine or placebo) and underwent chemotherapy at the same time.

In the same study, we did an analysis to investigate patients’ OS who received only interventions (BCc1 nanomedicine or placebo) without undergoing chemotherapy.

**Fig. 7:**
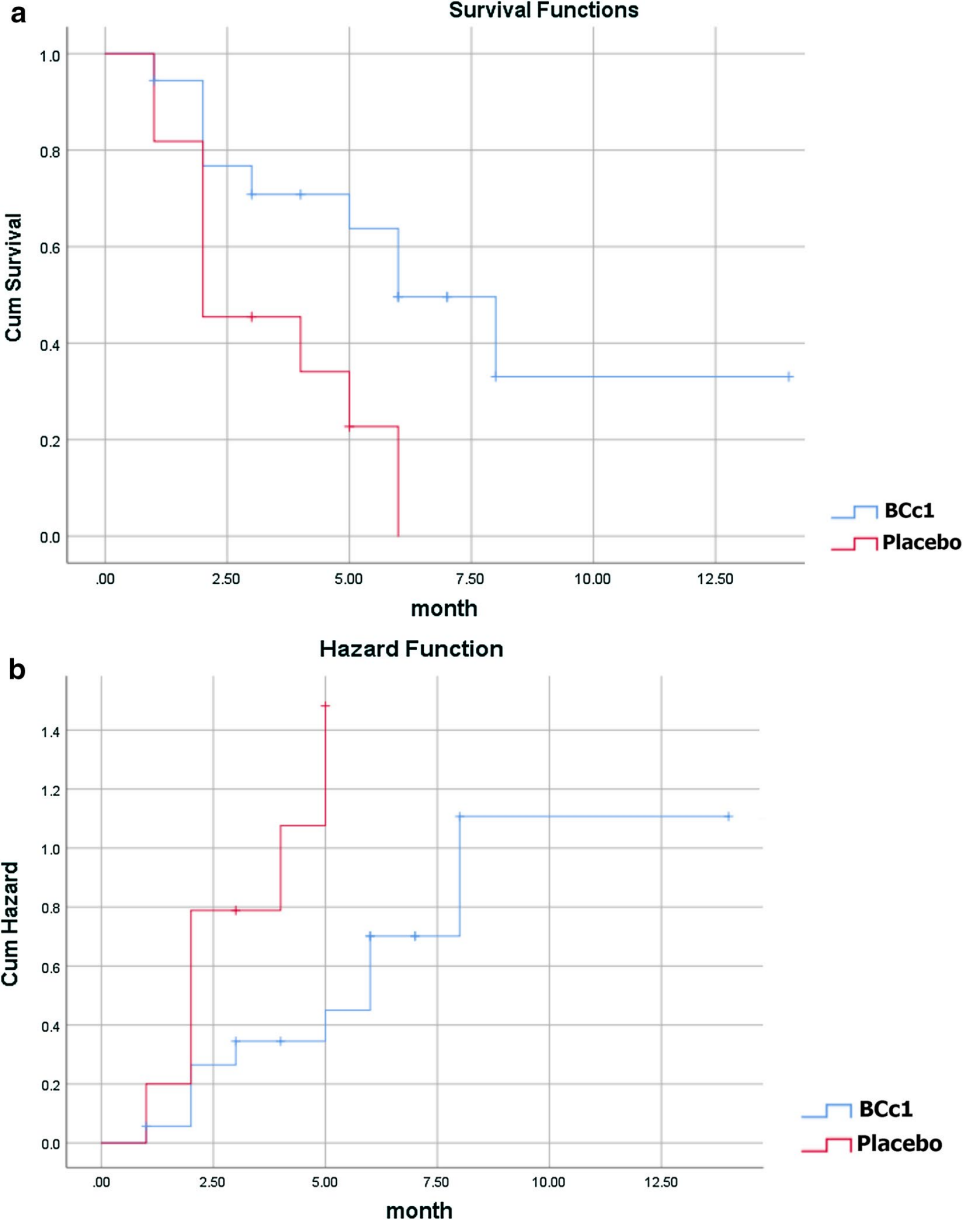
**a** Kaplan–Meier diagram of survival. **b** Hazard function diagram in metastatic patients who only received interventions (BCc1 nanomedicine or placebo) without undergoing chemotherapy

As shown in Table 5A, the results revealed that in metastatic patients who only took BCc1 capsules, the median OS was 122 days significantly longer than the median OS of those who received only placebo capsules. Kaplan–Meier diagram of survival and hazard function diagram of metastatic patients who received only intervention (BCc1 nanomedicine or placebo) are shown in Fig. 7a, b.

**Table 5:** Median of overall survival analysis of patients who only received intervention: (A) BCc1 nanomedicine or Placebo without chemotherapy. (B) Death percentage analysis of patients who only received intervention: BCc1 nanomedicine or Placebo without chemotherapy

Group	Metastatic		Non-metastatic	
Total number	BCc1(n = 19)	Placebo (n = 11)	BCc1(n = 18)	Placebo (n = 10)
*(A) Median of overall survival analysis*				
Median (days)	174	52	442	454
Std. error	46.75	46.64	41.43	44.635
Median 95% CI (lower)	118.40	0	361	366
Median 95% CI (upper)	229.59	113.69	523	541
P-value for test of equality of survival distributions	0.042		0.053	
HR (BCc1/Placebo)	0.500		0.324	
Stage (n)				
I	0	0	3	1
II	0	0	3	4
III	0	0	8	3
IIII	19	11	0	0
*(B) Death rate analysis median of overall survival analysis*				
Death rate (%)	49	63	13	30

Test of equality of survival distributions for (Bcc1 an0d placebo groups) assay with Log Rank (Mantel-Cox)

**Fig. 8:**
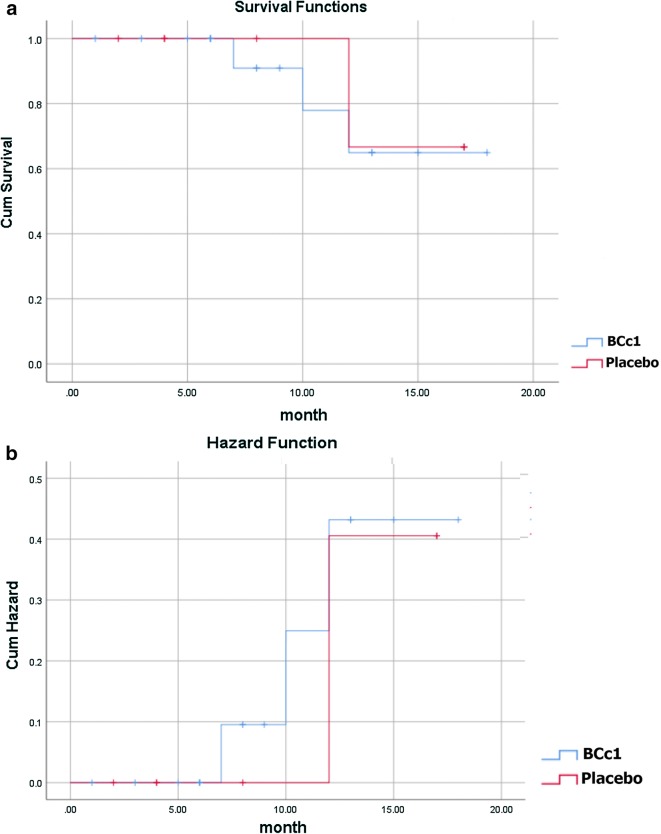
**a** Kaplan–Meier diagram of survival. **b** Hazard function diagram in non-metastatic patients who only received interventions (BCc1nanomedicine or placebo) without undergoing chemotherapy

On the other hand, as shown in Table 5A, in non-metastatic patients who only took placebo capsules, the median OS was 12 days longer, although not significantly, than the median OS of those who received BCc1. Kaplan–Meier diagram of survival and hazard function diagram of non-metastatic patients who received only intervention (BCc1 nanomedicine or placebo) are shown in Fig. 8a, b.

As shown in Table 5B, in metastatic patients, 49% of patients in BCc1 nanomedicine and 63% in placebo, and in non-metastatic patients, 13% in BCc1 nanomedicine and 30% in placebo died during the study, while patients of these groups received only the medicine (BCc1 nanomedicine or placebo), without undergoing chemotherapy.

The results of Cox multivariate regression model are shown in Table 6. In part A of Table 6, HR of BCc1 nanomedicine equals 0.476 [95% CI 0.24–0.96; p = 0.039]. This value of HR indicates that in metastatic patients, the hazard of mortality for those who took BCc1 nanomedicine is 0.476 times more than that of those who took placebo. Likewise, in part A of Table 6, HR of chemotherapy equals 0.737 [95% CI 0.37–1.47; p = 0.388]. This value of HR indicates that in metastatic patients, the hazard of mortality for those who underwent chemotherapy is 0.737 times more than that of those who did not receive chemotherapy.

**Table 6:** Cox multivariate regression model. (A) metastatic gastric cancer patients. (B) Non-metastatic gastric cancer patients

Variables in the equation						
	B	df	Sig.	Exp(B)	95.0% CI	
					Lower	Upper
*(A) Cox multivariate regression model for metastatic gastric cancer*						
BCc1 nanomedicine indicator	− 0.743	1	0.039	0.476	0.235	0.962
Chemotherapy indicator	− 0.306	1	0.388	0.737	0.368	1.474
*(B) Cox multivariate regression model for non*-*metastatic gastric cancer*						
BCc1 nanomedicine indicator	− 1.316	1	0.057	0.268	0.069	1.042
Chemotherapy-indicator	0.401	1	0.535	1.494	0.420	5.315

As shown in part B of Table 6, HR of BCc1 equals 0.268 [95% CI 0.07–1.04; p = 0.057]. This value of HR indicates that in non-metastatic patients, the hazard of mortality for those who took BCc1 nanomedicine is 0.268 times more than that of those who took placebo. Likewise, in part B of Table 6, HR of chemotherapy equals 1.494 [95% CI 0.42–5.32; p = 0.535]. This value of HR indicates that in non-metastatic patients, the hazard of mortality for those who underwent chemotherapy is 1.494 times more than that of those who did not receive chemotherapy.

### Adverse effects

The adverse effects including vomiting, nausea, neutropenia, and anorexia were not observed in patients in each treatment arm.

During the follow-up, no diarrhea was observed and 15 non-metastatic patients in BCc1 claimed an increase in appetite and also there were no adverse events leading to death in placebo and BCc1 nanomedicine. The results of the whole blood, renal function, biochemical results, and hepatic function are shown at Table 7A–D respectively.

**Table 7:** Whole blood (A), renal function (B), biochemical results (C) and hepatic function (D) of the patients who took BCc1 nanomedicine revealed that none of the patients suffered deficiency

Index	Unit	Amount
*(A) Whole blood function*		
WBC	×1000/mm^3^	5.1 ± 1.3
RBC	Mill/mm^3^	4.4 ± 0.6
HGB	g/dl	12.5 ± 1.8
HCT	%	40.1 ± 4.5
MCV	Fl	88.4 ± 10.2
MCH	pg	28.8 ± 4.3
MCHC	g/dl	32.9 ± 1.8
Platelet	×1000/mm^3^	195 ± 27
RDW		43 ± 21.1
PDW	Fl	13.3 ± 0.8
MPV	Fl	10 ± 1.6
*(B) Renal function*		
Urea	mg/dl	30.4 ± 13.6
Creatinine	mg/dl	1.0 ± 0.1
*(C) Biochemical function*		
Iron	µg/dl	56 ± 15
TIBC	µg/dl	314 ± 30
UIBC	µg/dl	258 ± 10
Ferritin	ng/dl	250 ± 80
Sodium	mmol/l	139 ± 7
Potassium	mmol/l	4.1 ± 0.7
Chloride	mmol/l	105 ± 10
*(D) Hepatic function*		
Triglycerides	mg/dl	102 ± 48
Total cholesterol	mg/dl	183 ± 22
AST (SGOT)	U/l	19.1 ± 5.1
ALT (SGPT)	U/l	14.3 ± 4.3
Alkaline phosphatase	IU/l	286.6 ± 95
Bilirubin (total)	mg/dl	0.725 ± 0.09
Bilirubin (direct)	mg/dl	0.2 ± 0.1

### Quality of life

The global life quality of all patients was analyzed based on EORTC QLQ-C22 form including dysphagia scale, eating restrictions scale, anxiety scale, dry mouth, taste, and hair loss topics and in this form, lower scores indicated better global QOL.

Before randomization, all patients filled out EORTC QLQ-C22 form once, saved as “before form” in patients’ documents. In patients who received the treatment, the form was filled out once more after 1.5 and 4 months for metastatic and non-metastatic patients, respectively, saved as “after form” in patients’ documents.

As shown in Table 8, the mean difference (before-after) of global QOL improved (3.25, p > 0.05) in metastatic patients who received BCc1, but it became worse (− 4.421, p < 0.05) in patients who received placebo. Likewise, as shown in Table 9, the mean difference (before-after) of global QOL improved (2.29, p > 0.05) in non-metastatic patients who received BCc1, but it became worse (− 3, p < 0.05) in patients who received placebo.

**Table 8:** (A) Quality of life of metastatic gastric cancer patients, (B) Median difference before–after of quality of Life

	Mean (before)	Std. deviation	Std. error	P-value	Mean (after)	Std. deviation	Std. error	P-value
*(A) Quality of life in patients with metastatic gastric cancer*								
Global quality (BCc1 nanomedicine)	48.88	13.614	2.779	0.118	45.63	11.009	2.247	0.200
Global quality (Placebo)	47.16	13.280	3.047	0.200	51.58	12.384	2.841	0.200

	Mean*	Std. deviation	Std. error mean	P-value
*(B) Median difference (before*–*after) of quality of life*				
Quality of BCc1 (before–after)	3.250	8.975	1.832	0.089
Quality of Placebo (before–after)	− 4.421	6.947	1.594	0.013

* Higher score show better global quality of life

**Table 9:** (A) Quality of life of non-metastatic gastric cancer patients, (B) Median difference before–after of quality of Life

	Mean (before)	Std. deviation	Std. error mean	P-value	Mean (after)	Std. deviation	Std. deviation	P-value
*(A) Quality of life in patients with non*-*metastatic gastric cancer*								
Global quality (BCc1 nanomedicine)	40.75	9.013	1.840	0.118	38.46	9.532	9.013	0.200
Global quality (Placebo)	47.11	12.009	2.831	0.200	50.11	14.046	12.009	0.200

	Mean*	Std. deviation	Std. error mean	P-value
*(B) Median difference (before*–*after) of quality of life*				
Quality of BCc1 (before–after)	2.29	5.497	1.122	0.053
Quality of Placebo (before–after)	− 3	12.009	2.831	0.038

* Higher score show better global quality of life

In the same study, we did an analysis to investigate patients’ QOL who received intervention (BCc1 or placebo) and chemotherapy simultaneously.

As shown in Table 10, the mean difference (before-after) of global QOL improved (1.66, p > 0.05) in metastatic patients who received BCc1 and chemotherapy simultaneously, but it became worse (− 0.75, p > 0.05) in patients who received placebo and chemotherapy at the same time. Likewise, as shown in Table 11, the mean difference (before-after) of global QOL improved (0.714, p > 0.05) in non-metastatic patients who received BCc1 and chemotherapy simultaneously, but it became worse (− 3.83, p < 0.05) in patients who received placebo and chemotherapy at the same time.

**Table 10:** (A) Quality of life of metastatic gastric cancer, (patient’s received intervention and chemotherapy simultaneously). (B) Median difference before –after of Quality of Life

	Mean (before)	Std. deviation	Std. error	P-value	Mean (after)	Std. deviation	Std. error	P-value
*(A) Quality of life in patients with metastatic gastric cancer*								
Global quality (BCc1 nanomedicine)	45.89	12.869	4.290	0.200	44.22	10.721	3.574	0.200
Global quality (Placebo)	49.08	13.318	3.844	0.200	49.83	13.265	3.829	0.200

	Mean*	Std. deviation	Std. error mean	P-value
*(B) Median difference (before*–*after) of quality of life*				
Quality of BCc1 (before–after)	1.667	7.053	2.351	0.499
Quality of Placebo (before–after)	− 0.750	2.958	0.854	0.399

* Higher score show better global quality of life

**Table 11:** (A) Quality of life of non- metastatic gastric cancer, (patient’s received intervention and chemotherapy simultaneously). (B) Median difference before–after of quality of life

	Mean (before)	Std. deviation	Std. error	P-value	Mean (after)	Std. deviation	Std. deviation	P-value
*(A) Quality of life in patients with non*-*metastatic gastric cancer*								
Global quality (BCc1 nanomedicine)	38.29	10.045	3.797	0.200	37.57	11.267	4.259	0.200
Global quality (Placebo)	50.75	10.931	3.155	0.200	54.58	12.176	3.515	0.200

	Mean*	Std. deviation	Std. error mean	P-value
*(B) Median difference (before*–*after) of quality of life*				
Quality of BCc1 (before–after)	0.714	6.184	2.337	0.077
Quality of Placebo (before–after)	− 3.833	5.441	1.571	0.033

* Higher score show better global quality of life

## Discussion

Scientists all around the world have discovered a human genome map by mixing forces, which has had a dramatic effect on production of current medicine. It has also broadened the views leading to advancement of making new drugs and biological agents [30]. These new drugs, despite humane genomes’ variability, must be capable to overcome various and special mechanisms causing diseases in every individual person.

Nanotechnology provides new approaches aiming for reduction of adverse effects of systemic delivery and improvement of efficacy [14, 31]. Nowadays, due to the heavy side effects of normal tissues in cancer therapy, produced by a conventional therapeutic dose of anti-tumor drugs, scientists are inclined to use targeted nano-drug-delivery systems [30].

The results of an animal study, conducted by Chen LC, revealed that intravenous administration of (188) Re-liposome could be beneficial and is considered a useful approach to delivering passive nano-targeted radio therapeutics in oncology applications [32].

Doxil^®^ (liposomal doxorubicin) in 1995 and Onivyde^®^ (liposomal irinotecan) in 2015 were the first generation of nanomedicine receiving clinical approval in the last two decades [33, 34].

The results of a meta-analysis disclosed that liposomal doxorubicin-based chemotherapy is associated with a significant reduction in the risk of cardio toxicity [35]. The FDA recently approved that nano liposomal irinotecan combined with fluorouracil/folinic acid can significantly prolong progression of free survival and OS in comparison to fluorouracil/folinic acid [36].

The nanochelating technology, patented [15] as a new approach, is proving to have a significant role in treatment of different diseases by synthesizing different nanostructures that possess effective properties. The very same nanostructures have some physical and chemical properties such as saturation solubility, dissolution rate, crystal form, particle surface hydrophobicity, and hydrophobicity physical response [37].

Nano particles that are produced by nanochelating technology are biodegradable and/or biocompatible and are synthesized using controlled self-assembly method [38]; as a result, the nanostructures produced by this method are completely unique because it is so powerful in chemistry protocols and can synthesize organic and inorganic building blocks into unprecedented structures and patterns [10].

In the previous study, BCc1 nanomedicine was designed based on the nanochelating technology for cancer treatment experimented in vitro and vivo studies and the results showed that BCc1 has high potentials to induce therapeutic behavior [8].

According to some data in 2013–2015, almost 0.8% of people will be diagnosed with stomach cancer at some point in their lifetime, so we investigated the effects of this nanomedicine in patients with gastric cancer [2].

BCc1 is the only nanomedicine that is designed and used in the domain of human cancers based on self-assembly method [38, 39]. Even in the most interiors of cancer stem cells (CSC) niches, nanomedicines are capable of delivering drug molecules and transcending the limitations of conventional free drug delivery approaches [40].

Most drug-resistant cancer cells and CSCs show high levels of CD44 receptor which binds hyaluronate [41]. CD44 is potentially able to be targeted for the treatment of drug-resistant tumors and CSC [42]. The results of the previous study revealed that BCc1 can reduce CD44 expression in cancer cell line; as a result, we can assume that BCc1 is able to affect CSCs [8].

One of the properties of BCc1 is chelating iron which can arrest G1/S phase and cause the death of cancer cells using apoptosis mechanism, while no apoptosis was observed in normal cells with an identical BCc1 dose [8]. In the present study, BCc1 nanomedicine does not have any side effects like nausea, diarrhea, vomiting, and anomie in patients with gastric cancer.

OS could be considered the primary endpoint of cancer treatment in the present and future [29]. BCc1 has already proved that it is able to increase the survival rates and decrease metastasis in mice bearing adenoma carcinoma. In the present study, in order to investigate the effect of BCc1 nanomedicine on OS at a clinical trial (randomized and placebo controlled), some patients with metastatic and none-metastatic gastric cancer were analyzed after taking BCc1 nanomedicine. It was shown that BCc1 nanomedicine can increase median OS compared to placebo, both in metastatic and none-metastatic gastric cancer patients.

As shown in Kaplan–Meier diagram for metastatic patients, half of the deaths in patients who took placebo capsules occurred in the first 30 days of the study, but half of the deaths in patients who took BCc1 nanomedicine occurred in the first 50 days (Fig. 3). In addition, as shown in Kaplan–Meier diagram for non-metastatic patients, half of the deaths in patients who took placebo capsules occurred in the first 3 months of the study, but half of the deaths in patients who took BCc1 nanomedicine occurred in the first 6 months (Fig. 4).

In conclusion, in both metastatic and non-metastatic patients, those who took BCc1 nanomedicine lived longer than those who took placebo.

As confirmed by ethics committee, no treatment was removed from the study and a number of patients received chemotherapy simultaneously both in BCc1 nanomedicine and placebo groups. To investigate the simultaneous effect of chemotherapy and intervention (BCc1 or placebo) on OS, an analysis was conducted on the data revealing that in patients with metastatic gastric cancer, the median OS was significantly longer, in patients who simultaneously received chemotherapy and BCc1 than those who received chemotherapy and placebo at the same time. The analysis also showed that in patients with none-metastatic gastric cancer, the median OS was longer, although not significantly, in patients who simultaneously received chemotherapy and BCc1 nanomedicine than those who simultaneously received chemotherapy and placebo.

These results prove the hypothesis that there is synergism effect between mechanisms of BCc1 nanomedicine and conventional treatments like chemotherapy.

To investigate the effect of BCc1 nanomedicine on OS, an analysis was conducted on the data revealing that in patients with metastatic and non-metastatic gastric cancer, those who only received BCc1 without chemotherapy lived longer, which shows the therapeutic effect of BCc1 nanomedicine. This finding can be the subject of future clinical trials with a larger number of patients.

QOL measures provide helpful information of global well-being and functional status of patients, which is why QOL is critical for complete evaluation of new treatments for patients with cancer [29, 43].

The study findings revealed that the BCc1 group enjoyed more improvement, regarding global QOL, in comparison with the placebo group in both metastatic and none-metastatic groups.

As the results show, the similar QOL and OS improvement pattern proves the positive effect of BCc1 nanomedicine on these patients.

A potential limitation of the trial was the point that because the patients suffered gastric cancer at metastatic stage, taking 3 capsules in each serving was hard for them; therefore, in the future, it is recommended to use BCc1 nanomedicine for gastric cancer patients (end stage and metastatic) through other application methods.

## Conclusion

The findings of the present study reveals that BCc1 nanomedicine could have a positive effect on QOL and OS without any side effects in patients with cancer in addition to its therapeutic effects as a medicine alone and also BCc1 nanomedicine could have synergism effects when used with chemotherapy drugs at the same time. Complementary studies in the future would hopefully confirm the effectiveness of our new nanomedicine for cancer treatment.

## Additional file

**Additional file 1.** BCc1 nanomedicine toxicity report (Oral LD50 = 1776.59 mg/kg).

## Abbreviations

FDA: Food and Drug Administration
IP: intraperitoneal
LD50: lethal dose
OS: overall survival
QOL: quality of life
EUS: endoscopic ultrasound
CT scan: computed tomography scan
PET scan: positron emission tomography scan
AJCC: American Joint Committee on Cancer staging system guideline
NOAEL: No Observed Adverse Effect Level
FIH: first-in-human
API: active pharmaceutical ingredient
HRs: hazard ratios
CI: confidence interval
CSC: cancer stem cells
