# Supplementary figures and images for: A randomized, double-blind, placebo-controlled investigation of BCc1 nanomedicine effect on survival and quality of life in metastatic and non-metastatic gastric cancer patients
Source: J Nanobiotechnology. 2019 Apr 10;17:52. doi: 10.1186/s12951-019-0484-0 (PMC6458717; doi:10.1186/s12951-019-0484-0)

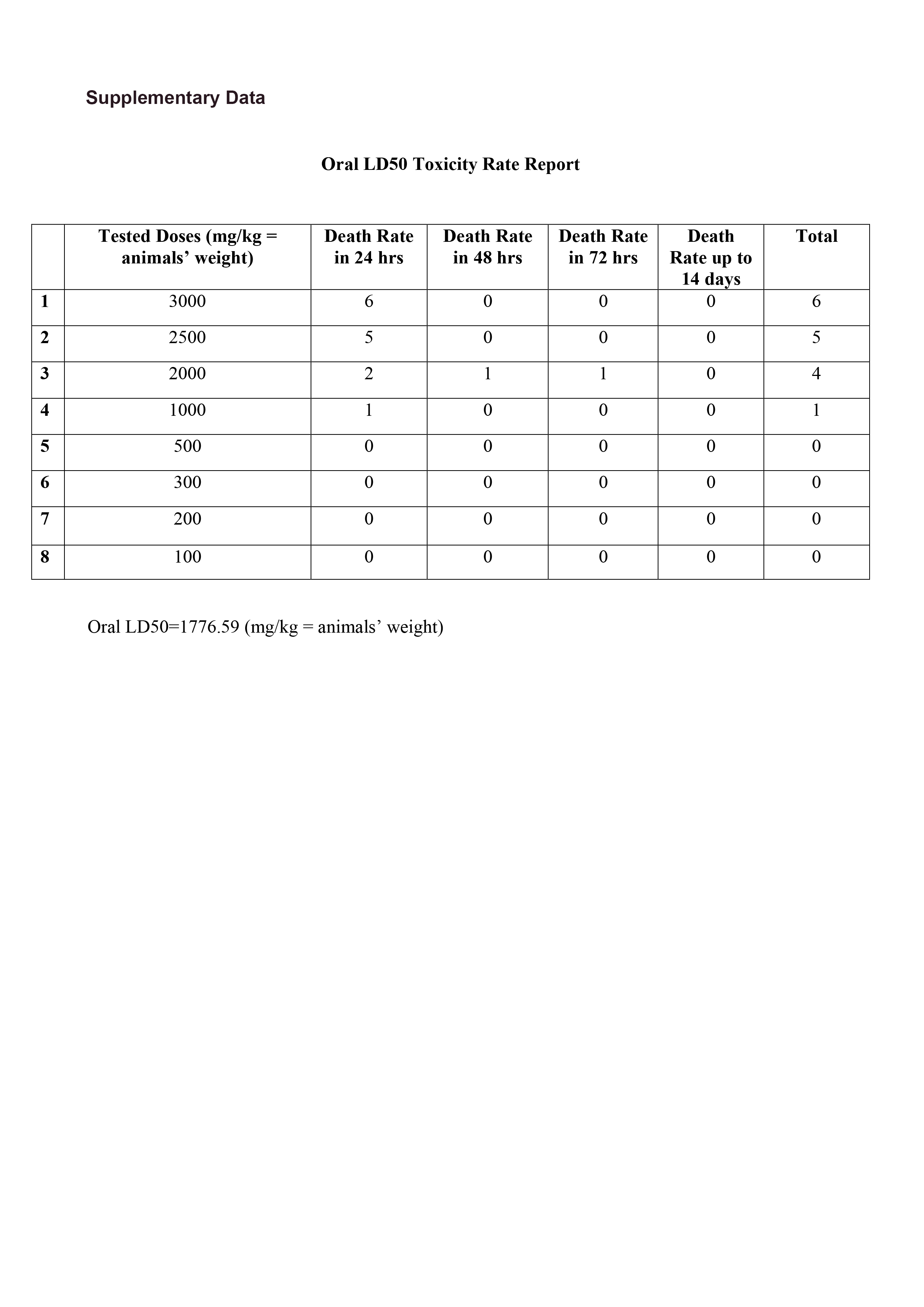

Supplement: Supplementary file 1 — Additional file 1. BCc1 nanomedicine toxicity report (Oral LD50 = 1776.59 mg/kg). [file 12951_2019_484_MOESM1_ESM.jpg]
